# Antimicrobial Peptides: An Emerging Hope in the Era of New Infections and Resistance

**DOI:** 10.3390/antibiotics14060546

**Published:** 2025-05-27

**Authors:** Piyush Baindara

**Affiliations:** Animal Science Research Center, Division of Animal Sciences, University of Missouri, Columbia, MO 65211, USA; piyush.baindara@gmail.com

Recently, antimicrobial peptides (AMPs) have garnered significant attention as a viable alternative to traditional antibiotics. AMPs are naturally occurring important elements of the host defense system that are functional across all biological domains, from prokaryotes to eukaryotes [1]. AMPs are powerful antimicrobial agents exhibiting a wide range of biological activity against infectious and pathogenic entities, including bacteria, fungi, viruses, and parasites [2]. Furthermore, the immuno-modulatory and potential anticancer properties of AMPs have been well reported [3].

Interestingly, AMPs are extremely diverse, thus leading to endless possibilities for new variants. The human gut microbiota is one such example of a complex system that is vital to human health due to its diversity and dynamic competition [4]. Notably, gut AMPs work synergistically with other gut microbiota and antimicrobials to maintain gut homeostasis. Additionally, gut AMPs are evolving under complicated and highly synergistic co-evolutionary pressure developed by interactions between various competitive microbiota and their respective AMPs [5]. The synergistic actions of gut AMPs with conventional antibiotics have been suggested as a key weapon to fight against multi-antibiotic-resistant bacteria [6]. In a recent study, Pandey et al. reported multi-antibiotic resistance in different Vibrio species recovered from environmental water samples [7]. This suggests that antibiotic disposal in the environment is also triggering the emergence of drug resistance not only competition or direct antibiotic intake.

AMPs have been considered one of the potential alternatives to fight against multi-drug-resistant bacteria, and several are undergoing clinical trials [8]. Similarly, a new AMP, NNS5-6, produced by mangrove bacteria *Paenibacillus thiaminolyticus* NNS5-6, has displayed antimicrobial activity against drug-resistant *Pseudomonas aeruginosa* and *Klebsiella pneumonia* [9]. Furthermore, recombinant mussel adhesive proteins fused with functional peptides (MAP-FPs) have been characterized and exhibit specific activity against Gram-negative bacteria such as *Escherichia coli*, *Salmonella typhimurium*, and *K. pneumonia* [9]. Notably, MAP-FPs were found to be nontoxic against mammalian cell lines, suggesting they are suitable candidates for therapeutic applications.

Natural AMP-inspired synthetic peptides are one strategy to achieve highly efficient AMPs with low toxicity to combat drug-resistant pathogens in therapeutic settings [10]. Additionally, synthetic peptides and their truncated forms facilitate the easy examination and characterization of antimicrobial efficacy to design better AMPs against drug-resistant pathogens [11]. In a recent study, Meier et al. examined and demonstrated the antimicrobial potential of a synthetic peptide, C18G, and its several truncated forms using model lipid membranes and vesicles. The findings suggest that peptide length and ensuing hydrophobic matching are critical factors to consider in the evolution and design of membrane-disrupting AMPs [12]. Moreover, Keeratikunakorn et al. synthesized a natural AMP, BiF2_5K7K, using amino acid substitution based on residue composition and distribution. BiF2_5K7K displayed superior activity against both Gram-positive and Gram-negative bacteria isolated from boar semen and sow vaginal discharge. Interestingly, BiF2_5K7K treatment achieved a superior pregnancy and farrowing rate in an artificial insemination test at a pig farm [13]. Similarly, synthetic AMPs A-11 and AP19 were reported to restrict the growth of Gram-negative bacteria isolated from fresh and stored boar semen at 18 °C while not harming sperm motility, acrosomal integrity, and viability [14]. Overall, synthetic AMPs, BiF2_5K7K, A-11, and AP19 could be potential alternatives to conventional antibiotics for use in boar semen extenders.

Other than direct antimicrobial activity, AMPs engage in immunomodulatory activities, thus shaping the outcomes of antimicrobial therapies. Finkina et al. reported the immunomodulatory effects of a tobacco defensin, NaD1, on human macrophages, dendritic cells, and bold monocytes. Also, NaD1 could induce both pro-inflammatory and anti-inflammatory cytokines, suggesting its possible potential for therapeutic applications [15]. Another synthetic AMP, MV6, showed synergistic interactions and an aminoglycoside netilmicin. Interestingly, MV6 lacks intrinsic antimicrobial activity; however, it reduces mutant prevention concentration of an aminoglycoside netilmicin against *Acinetobacter baumannii* when used synergistically [16]. Conclusively, the development of synthetic AMPs based on natural AMPs or by using special algorithms has the potential to generate more efficient and specific AMPs to fight the battle against drug resistance. Slavokhotova et al. developed an algorithm for the prediction of α-hairpins based on characteristic motifs containing four or six cysteines. There were more than 2000 putative α-hairpins predicted, and the authors concluded that AMPs containing six cysteines had more potent antimicrobial activity than the AMPs with four cysteines [16]. Interestingly, AMPs containing cysteine motifs are evolutionarily conserved in all domains of life, ranging from prokaryotes to eukaryotes, which also suggests a potential evolutionary link and role with antimicrobial efficacy [17,18,19].

In conclusion, AMPs are promising drug candidates to combat drug-resistant pathogens. Notably, 12 peptide-based drugs with potential antimicrobial or antifungal properties have been approved by the US Food and Drug Administration (FDA) since 1955. As of now, several AMPs are undergoing clinical trials targeting drug-resistant pathogens [8]. Interestingly, rezafungin (a novel systemic antifungal), an echinocandin and a class of cyclic lipopeptides, was approved by the FDA in March 2023 [20]. Overall, AMPs are an emerging hope in the era of new infections and drug resistance (Figure 1). AMPs provide endless opportunities to discover new peptides, especially in the case of bacterial AMPs [21,22]. As bacterial diversity is wide, there are significant possibilities to discover new AMPs; in fact, more than 99% of bacterial diversity is still unexplored [23]. Additionally, complex biological systems such as skin, the lungs, and gut microbiota are highly competitive environments that have favorable conditions for the production of new AMPs [24,25,26]. Also, recent scientific advances along with artificial intelligence (AI) have contributed exceptionally to the discovery of novel unexplored AMPs from different domains of life, including the extinct ones [27,28]. Recently, AMPs have been extensively explored as an alternative to conventional antibiotics for therapeutic applications; however, there is a long way to go before AMPs can be fully adopted in clinical settings, as there are many unanswered questions about AMPs that require further detailed study [29].

- How do AMPs selectively target microbes over host cells? Can this selectivity be manipulated synthetically?
- How do the AMP producers maintain self-immunity?
- Is it possible to develop resistance against AMPs? If so, what are the AMP resistance genes and what is the global prevalence? Can it be transferred horizontally like conventional antibiotics?
- How do repeated AMP treatments influence microbial diversity?
- How does AMP treatment influence pro- and anti-inflammatory immune response?
- What signaling pathways are activated upon AMP–host cell interactions?

## Figures and Tables

**Figure 1:**
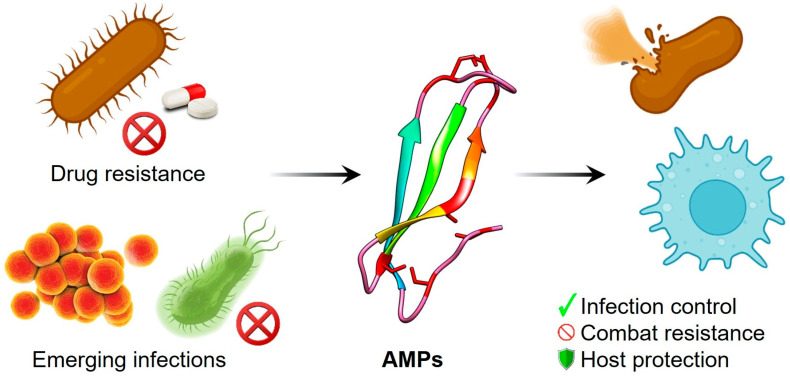
AMPs combating drug resistance and infection.
